# C5aR1 promotes acute pyelonephritis induced by uropathogenic *E*. *coli*

**DOI:** 10.1172/jci.insight.97626

**Published:** 2017-12-21

**Authors:** Ke Li, Kun-Yi Wu, Weiju Wu, Na Wang, Ting Zhang, Naheed Choudhry, Yun Song, Conrad A. Farrar, Liang Ma, Lin-lin Wei, Zhao-Yang Duan, Xia Dong, En-Qi Liu, Zong-Fang Li, Steven H. Sacks, Wuding Zhou

**Affiliations:** 1 Core Research Laboratory, The Second Affiliated Hospital, School of Medicine, Xi’an Jiaotong University, Xi’an, China.; 2 Medical Research Council (MRC) Centre for Transplantation, King’s College London, Guy’s Hospital, United Kingdom (UK).; 3 Department of Nephrology, The Second Affiliated Hospital, School of Medicine, Xi’an Jiaotong University, Xi’an, China.; 4 Research Institute of Atherosclerotic Disease, School of Medicine, Xi’an Jiaotong University, Xi’an, China.; 5 National Local Joint Engineering Research Centre of Biodiagnostics and Biotherapy, The Second Affiliated Hospital, Xi’an Jiaotong University, Xi’an, China.

**Keywords:** Nephrology, Complement, UTI/pyelonephritis

## Abstract

C5a receptor 1 (C5aR1) is a G protein–coupled receptor for C5a and also an N-linked glycosylated protein. In addition to myeloid cells, C5aR1 is expressed on epithelial cells. In this study, we examined the role of C5aR1 in bacterial adhesion/colonization of renal tubular epithelium and addressed the underlying mechanisms of this role. We show that acute kidney infection was significantly reduced in mice with genetic deletion or through pharmacologic inhibition of C5aR1 following bladder inoculation with uropathogenic *E*. *coli* (UPEC). This was associated with reduced expression of terminal α-mannosyl residues (Man; a ligand for type 1 fimbriae of *E*. *coli*) on the luminal surface of renal tubular epithelium and reduction of early UPEC colonization in these mice. Confocal microscopy demonstrated that UPEC bind to Man on the luminal surface of renal tubular epithelium. In vitro analyses showed that C5a stimulation enhances Man expression in renal tubular epithelial cells and subsequent bacterial adhesion, which, at least in part, is dependent on TNF-α driven by C5aR1-mediated intracellular signaling. Our findings demonstrate a previously unknown pathogenic role for C5aR1 in acute pyelonephritis, proposing a potentially novel mechanism by which C5a/C5aR1 signaling mediates upregulation of carbohydrate ligands on renal tubules to facilitate UPEC adhesion.

## Abstract 2

C5aR1 plays a pathogenic role in acute pyelonephritis and facilitates bacterial colonization of epithelium.

## Introduction

Urinary tract infections (UTIs) remain among the most common infectious disease worldwide (1). It is frequent in women and children and is a particular problem for patients with diabetes, renal transplants, and catheterization (2–4). UTIs present as a wide spectrum of diseases, including bladder infection (cystitis), kidney infection (pyelonephritis), and kidney-infection-related complications such as renal scarring and sepsis. The causative organisms are usually uropathogenic strains of *E*. *coli* (UPEC). Despite antimicrobial therapy, the treatment of UTI remains a challenge for dealing with frequent recurrence or persistence of infection. Coupled with an increase in antibiotic resistance, even for organisms of confirmed antibiotic sensitivity (5, 6), this highlights a need to improve our understanding of the pathogenic mechanisms underlying this disease, in order to develop novel treatment strategies and improve current treatments.

Acute pyelonephritis (APN) resulting from bacterial infection of the renal parenchyma is an acute and severe kidney infection. The pathogenesis of APN can be influenced by the virulence of the infecting pathogens, the host-pathogen interaction, and host responses to pathogens, in addition to other factors such as urinary obstruction (7, 8). Most UPEC express a variety of fimbriae (e.g., P, Dr, and type 1) that contain lectins that enable them to bind to carbohydrate structures in glycoproteins and glycolipids on renal tract epithelial cells, which is a critical step in colonization (9). It has been suggested that P fimbriae recognize specific glycolipids [e.g., α-D-Gal-(1,4)-β-D-Gal] on the luminal surface of renal tubular epithelium and interact with Toll-like receptor 4 (TLR4), which elicits local inflammatory responses that promote tissue destruction and is associated with APN (10). In contrast to P fimbriae, Dr fimbriae recognize the receptors (e.g., CD55) located on peritubular basement membranes adjacent to the interstitium. This interaction does not cause significant tissue destruction, but is associated with chronic pyelonephritis (CPN) (11). Type 1 fimbriae are expressed on most UPEC strains and they recognize glycoproteins (e.g., mannosylated glycoproteins) on urinary tract epithelium. It has been suggested that type 1 fimbriae are essential for UPEC adhesion and colonization, thereby contributing to the development of UTI, particularly cystitis (12). Upon contact with epithelial cells, UPEC liberate toxins that mediate direct cellular injury, disrupting the mucosal barrier and providing access to the underlying tissue (13, 14). UPEC colonization and entry into underlying tissue can initiate innate immune responses, such as direct microbial killing by complement activation and microbial elimination by inflammatory cells. However, most human UPEC strains are resistant to complement-mediated killing (15, 16), and several lines of study have suggested that these bacteria mediate excessive inflammatory responses that cause epithelial destruction (17–19), impair innate immune cell function, and lead to persistent infection and renal fibrosis (20).

C5a is generated by the cleavage of complement component C5. C5a/C5aR1 interaction mediates a broad spectrum of proinflammatory reactions such as increased vascular permeability, recruitment of leukocytes to sites of injury or infection, generation of cytotoxic oxygen radicals (by granulocytes), and the production of proinflammatory mediators (21). A large body of research has demonstrated that C5a/C5aR1 signaling contributes to the pathogenesis of a wide range of inflammatory and immunological diseases (22, 23), but its participation in infectious disease is less known.

Our recent work in a murine model of CPN has shown that C5a/C5aR1 interactions play an important role in the pathogenesis of chronic renal inflammation and tubulointerstitial fibrosis (20). However, the role of C5aR1 in APN is presently unknown. Such information will improve our understanding of pathogenic roles of C5aR1 in ascending UTI and help to develop novel therapeutic strategies for improving treatment of acute UTI.

Bacterial–host cell carbohydrate interactions are known to facilitate tissue invasion. Terminal α-mannosyl residues (Man) are the carbohydrate ligand for type 1 fimbriae on UPEC (24, 25). Putative membrane proteins containing such ligands have been reported in the bladder (26). However, it is unknown whether such carbohydrate ligands are present within the kidney and whether they play a significant role in the development of pyelonephritis. Given that proinflammatory mediators have been suggested to play a role in regulation of cell surface mannosylated glycan expression (27), and that C5aR1 signaling regulates multiple cellular responses, we hypothesized that C5a/C5aR1 interactions mediate upregulation of carbohydrate ligands on renal tubules to facilitate UPEC adhesion/colonization that increases susceptibility to APN.

To address this, we employed a well-established murine model of APN, which is induced by bladder inoculation with human UPEC strain J96 (19, 28, 29). We applied this model to both wild-type (WT) and C5aR1-deficient mice (*C5aR1*^–/–^) to determine the pathogenic roles of C5aR1 in acute kidney infection. We performed a series of ex vivo (renal tissue) and in vitro (primary cultures of renal tubular epithelial cells [RTECs]) experiments to assess the impact of C5aR1 on Man expression and UPEC adhesion/colonization of renal tubular epithelium and RTECs. Bone marrow–chimera experiments were performed to evaluate the relative contributions of C5aR1 expressed on renal parenchyma or myeloid cells to the etiology of APN. C5aR1 antagonist (PMX53) was used to assess the therapeutic potential of targeting C5aR1 in our UTI model. Our data demonstrate a previously unknown pathogenic role for C5aR1 in APN and suggest a potentially novel mechanism by which C5aR1 mediates enhancement of Man-dependent bacterial colonization of renal tubular epithelium increasing susceptibility to ascending UTI.

## Results

### C5aR1 deficiency protects mice from APN.

**Figure 1:**
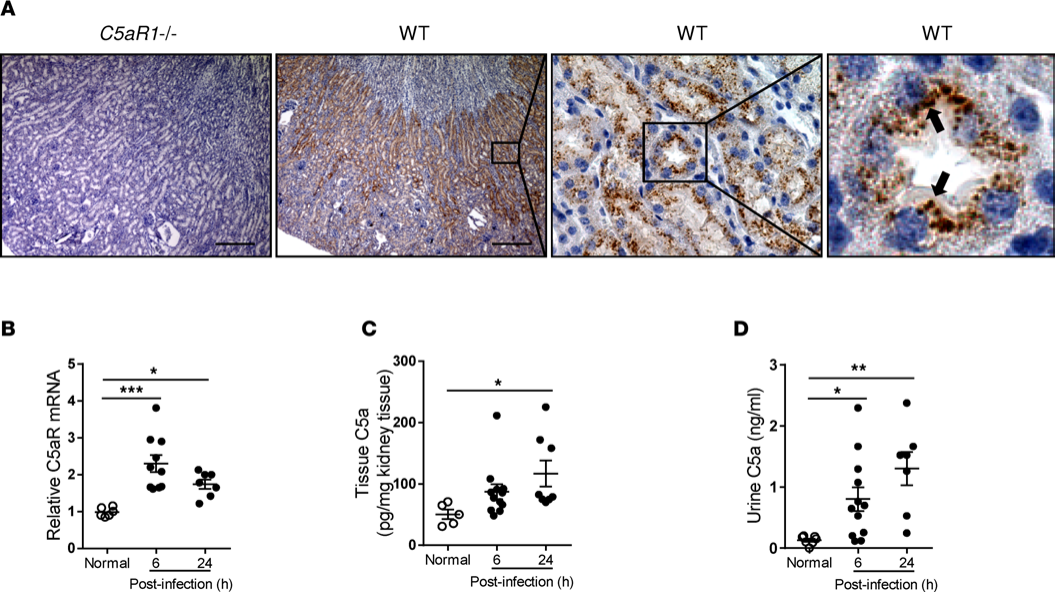
Detection of C5a and C5aR1 in urinary tract. (**A**) C5aR1 expression in WT kidney tissue determined by immunochemistry. Kidney tissue from *C5aR1^–/–^* mice was used as a negative control. Scale bars: 250 μm. Boxed regions correspond to the next (right) image (scale bars: 50 μm and 10 μm, respectively). Arrows indicate positive C5aR1 staining. A representative of 3 experiments is shown. (**B**) Relative mRNA levels of C5aR1 in normal and infected kidney tissues determined by RT-qPCR (*n* = 6 normal, *n* = 7–10 infected). (**C** and **D**) C5a levels in kidney tissue and urine samples determined by ELISA (*n* = 5–7 normal, *n* = 7–12 infected). (**B**–**D**) Each data point represents an individual mouse. Data were analyzed by 1-way ANOVA with Tukey’s multiple comparisons test. *P < 0.05, **P < 0.005, ***P < 0.001.

Although the expression of C5aR1 in the kidney has been previously reported (30, 31), distribution within the kidney has not been well documented. Therefore, we thoroughly examined the expression of C5aR1 in murine kidney. Our expression studies confirmed the presence of C5aR1 on the luminal surface of renal tubule cells with concomitant increase of C5aR1 mRNA expression in the kidney at 6 and 24 hours postinoculation (hpi) (Figure 1, A and B). C5a expression was detected in renal tissues and urine samples under normal conditions, which was elevated at 6 and 24 hpi (Figure 1, C and D). Thus, abundant expression of C5aR1 in renal tubular cells, upregulation of intrarenal C5aR1 expression, and elevation of C5a levels following infection support an involvement of C5a/C5aR1 in UTIs.

**Figure 2:**
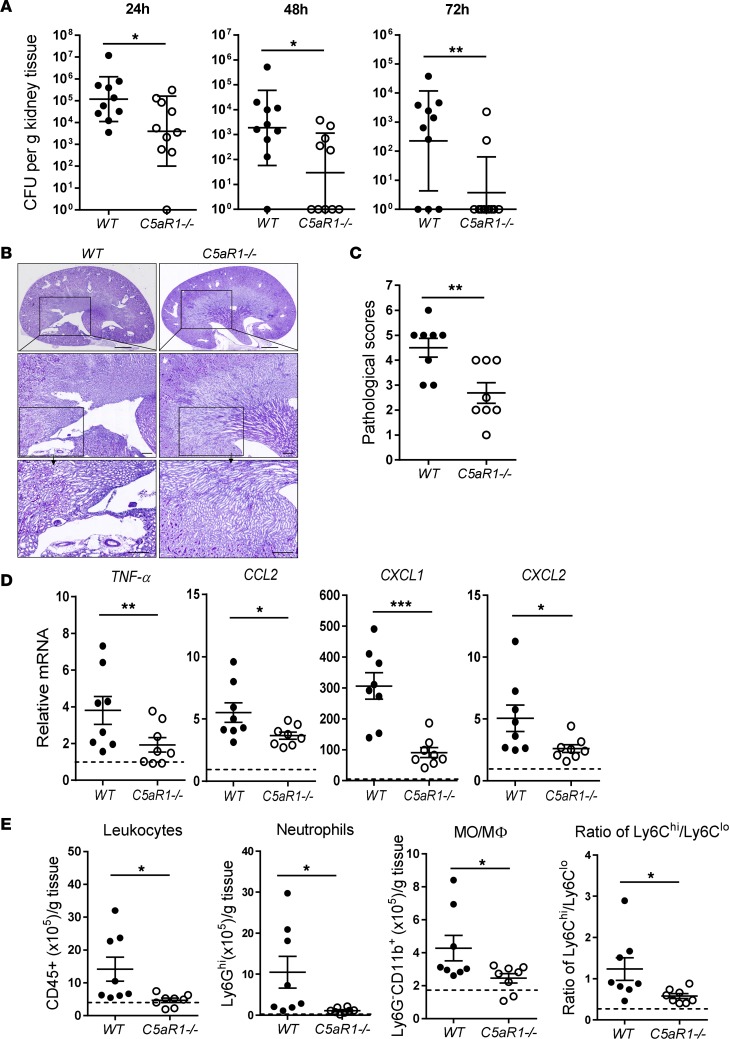
C5aR1 deficiency protects mice from acute pyelonephritis. (**A**) Bacterial loads in kidney tissues of WT and *C5aR1^–/–^* mice at 24, 48, and 72 hours postinfection (hpi) determined by bacterial plate count assay (*n* = 10/group). (**B**) Representative images of PAS staining of kidney sections of WT and *C5aR1^–/–^* mice at 72 hpi. Top panel: Cross-sections of the whole kidney. Scale bars: 1,000 μm. Middle and lower panels: Higher magnification images corresponding to the boxed regions in the above panel. Scale bars: 250 μm. (**C**) Histological scores of kidney sections of WT and *C5aR1^–/–^* mice at 72 hpi (*n* = 8/group). (**D**) Relative mRNA levels of proinflammatory mediators in infected kidney tissues of WT and *C5aR1^–/–^* mice at 6 hpi determined by RT-qPCR (*n* = 8/group). The dotted line across each graph represents the gene expression level of normal kidney tissue, which is similar between WT and *C5aR1^–/–^* mice. (**E**) Quantification of inflammatory cells in infected kidney tissues of WT and *C5aR1^–/–^* mice at 24 hpi. The dotted line across each graph represents the levels of inflammatory cells in normal kidney tissue, which is similar between WT and *C5aR1^–/–^* mice. MO/MΦ, monocytes/macrophages. For all data, each data point represents an individual mouse. Mann-Whitney test was used for CFU data in **A**. Unpaired 2-tailed Student’s *t* test was used for the data in **B**–**E**. *P < 0.05, **P < 0.005, ***P < 0.001.

To determine the role of C5aR1 in APN, we induced the infection in WT and *C5aR1*^–/–^ mice, and then assessed renal bacterial load and tissue destruction up to 72 hpi. *C5aR1*^–/–^ mice had significantly lower bacterial loads in the kidney at all time points (24, 48, and 72 hpi) (Figure 2A). Renal histopathological changes (i.e., cellular infiltration, bacterial patchiness, tubule destruction) within the cortical-medullar junction and the medulla were assessed as previously described (19). *C5aR1*^–/–^ mice displayed less severe renal histological lesions (72 hpi) when compared with WT controls (Figure 2, B and C). We also assessed tissue inflammation at early time points of postinoculation (6 hpi). Semiquantitative reverse transcription PCR (RT-qPCR) analysis revealed that intrarenal gene expression of key inflammatory mediators (i.e., *TNFA*, *CCL2*, *CXCL1*, *CXCL2*) were significantly reduced in *C5aR1*^–/–^ mice compared with WT controls (Figure 2D). Flow cytometric analysis showed that *C5aR1*^–/–^ mice had significantly fewer infiltrating leukocytes (i.e., total leukocytes, neutrophils, monocytes/macrophages [MO/MΦ], inflammatory monocytes) in the kidney, compared with WT controls (24 hpi) (Figure 2E and Supplemental Figure 1; supplemental material available online with this article; https://doi.org/10.1172/jci.insight.97626DS1).

Collectively, these data demonstrate that acute kidney infection was significantly reduced in *C5aR1^–/–^* mice, supporting a pathogenic role for C5aR1 in the development of APN.

### C5aR1 is required for Man expression in renal tubules and bacterial colonization of renal tract epithelium.

**Figure 3:**
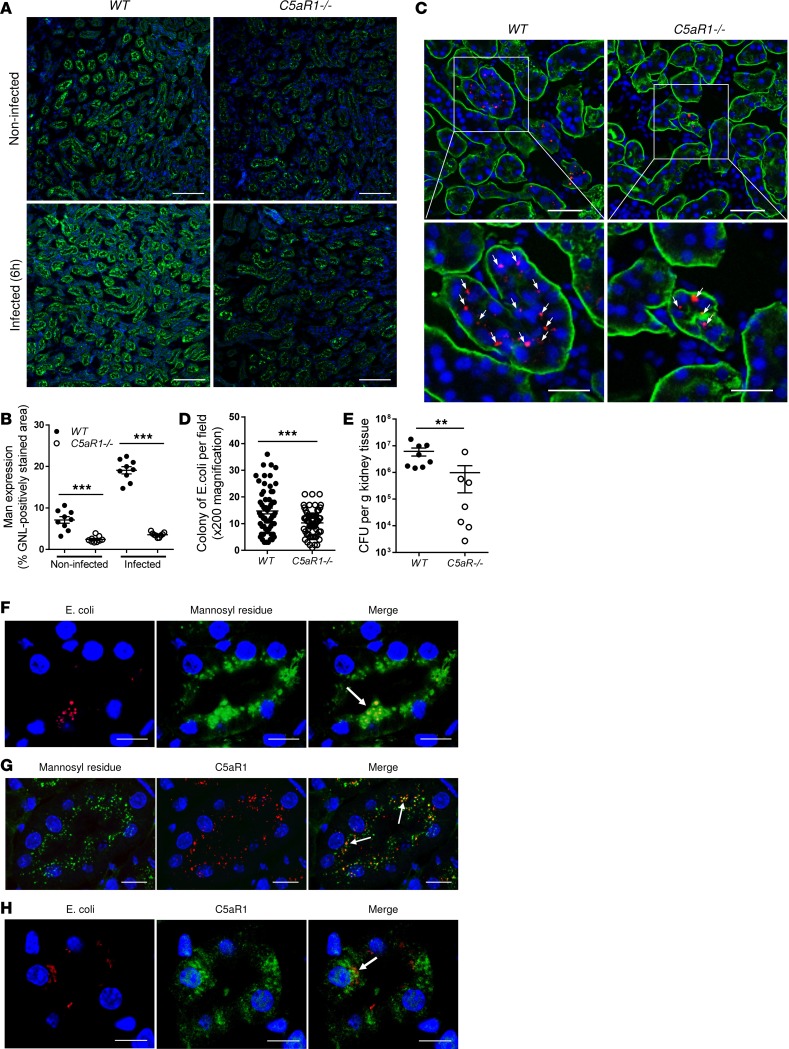
C5aR1 is required for mannosyl residue expression in renal tubules and bacterial colonization of renal tract epithelium (in vivo). (**A**) Representative images of mannosyl residue (Man) expression detected by fluorescein-labeled *Galanthus* *nivalis* lectin (GNL) (green) in kidney sections of WT and *C5aR1^–/–^* mice (i.e., noninfected and infected with J96 for 6 hours). Scale bars: 100 μm. (**B**) Quantification of Man expression in renal tubules corresponding to the WT and *C5aR1^–/–^* mice in **A**. Data were analyzed by 1-way ANOVA with Tukey’s multiple comparisons test (*n* = 9 viewing fields from 3 [normal] or 4 [infected] mice/group). ***P < 0.001. (**C**) Representative fluorescence microscopy images of kidney sections of WT and *C5aR1^–/–^* mice at 6 hours postinoculation (hpi) of TRITC-labeled J96 (red), proximal tubular marker (L-fucose) detected by *Lotus* *tetragonolobus* lectin (LTL) (green), and nuclear marker DAPI (blue) are shown, demonstrating early bacterial colonization of renal tubular epithelium. Boxed regions correspond to the bottom images. Arrows indicate bacterial colonies. Scale bars: 50 μm (top) and 25 μm (bottom). (**D**) Quantification of bacterial colonies in renal tabular epithelium corresponding to the WT and *C5aR1^–/–^* mice in **C**. Data were analyzed by unpaired 2-tailed Student’s *t* test (*n* = 60 viewing fields from 8 mice/group, under ×200 magnification). ***P < 0.001. (**E**) Bacterial loads in kidney tissues corresponding to the WT and *C5aR1^–/–^* mice in **C** were also determined by bacterial plate count assay. Data were analyzed by Mann-Whitney test. **P < 0.01. In **A**–**E**, a representative of 2 experiments is shown. (**F**) Fluorescence microscopy images of binding of bacteria (red) to Man (green) at the luminal surface of renal tubular epithelium of WT mice following the inoculation of TRITC-labeled J96 (6 hpi). Scale bars: 10 μm. (**G**) Fluorescence microscopy images demonstrating the colocalization of C5aR1 (red) and Man (green) in renal tubules of WT mice. Scale bars: 20 μm. (**H**) C5aR1 immunofluorescence microscopy images of the renal tubule of WT mice at 6 hpi of TRITC-labeled J96, demonstrating the association of C5aR1 (green) and bacteria (red) in renal tubular epithelial cells. Scale bars: 10 μm. (**F**–**H**) A representative of 3 experiments is shown.

We first determined the expression of glycans within the kidney and its association with C5aR1 status, by performing lectin staining in both normal and infected kidneys. We employed 3 fluorescein-labeled lectin probes: *Galanthus* *nivalis* lectin (GNL, which binds to [α-1,3]Man); *Lens* *culinaris* agglutinin (LCA, which binds to branched fucose/mannose residues); and *Sambucus* *nigra* bark lectin (SNA, which binds to sialic acid attached to terminal galactose). These 3 lectins have been used for the detection of high-mannose, hybrid, and complex N-glycans, respectively. GNL staining clearly showed the presence of Man in normal kidneys, which was located mainly within the cortical and medullary tubules, with predominant expression on the luminal surface of tubular epithelial cells (Supplemental Figure 2). Interestingly, GNL staining was significantly reduced in renal tubules of *C5aR1^–/–^* kidneys when compared with WT controls, including both infected (6 hpi) and noninfected kidneys (Figure 3, A and B). However, LCA and SNA staining was predominantly localized to the basolateral surface of renal tubules, with no significant difference observed between WT and *C5aR1^–/–^* kidneys following UPEC inoculation (Supplemental Figure 3). These observations demonstrate that Man, the carbohydrate ligands for type 1 fimbriae, are present within the kidney, and absence of C5aR1 is specifically associated with a reduction of Man expression on the luminal surface of renal tubules.

We then determined whether the reduced Man expression is associated with UPEC colonization of renal tract epithelium. Fluorescence microscopy and bacterial plate count assay revealed that bacterial colonies either in the tubular epithelium or the kidney homogenate were significantly reduced in *C5aR1^–/–^* mice (6 hpi), compared with WT mice (Figure 3, C–E), suggesting that bacterial adhesion/colonization of the renal tract epithelium is C5aR1 dependent. In addition to the J96 strain, we also observed C5aR1-dependent bacterial colonization in other human pyelonephritis strains: IH11128 (lack of P fimbriae) and CFT073 (data not shown). We also investigated whether UPEC colonize renal epithelium by binding Man on renal tubular epithelium. Fluorescence microscopy images revealed colocalization of bacteria with Man on the luminal surface of renal tubules, indicating Man-dependent UPEC adhesion in the kidney (Figure 3F). In addition, we examined the spatial relationship between C5aR1 and either Man or bound bacteria in renal tubules by immunofluorescence microscopy. Both Man and C5aR1 were predominantly detected on the luminal surface of renal tubules; a colocalization, albeit partial, was observed between C5aR1 and Man (Figure 3G). Bacterial colonies were also closely associated with C5aR1 on the luminal surface of renal tubules of infected kidneys (Figure 3H). Hence, C5aR1 protein is closely associated with Man and bacterial adhesion to renal tubules.

Collectively, these findings support a dual requirement of C5aR1 for Man expression in renal tubules and colonization of renal tract epithelium by UPEC.

### C5a stimulation upregulates Man expression and enhances bacterial adhesion/invasion in RTECs.

**Figure 4:**
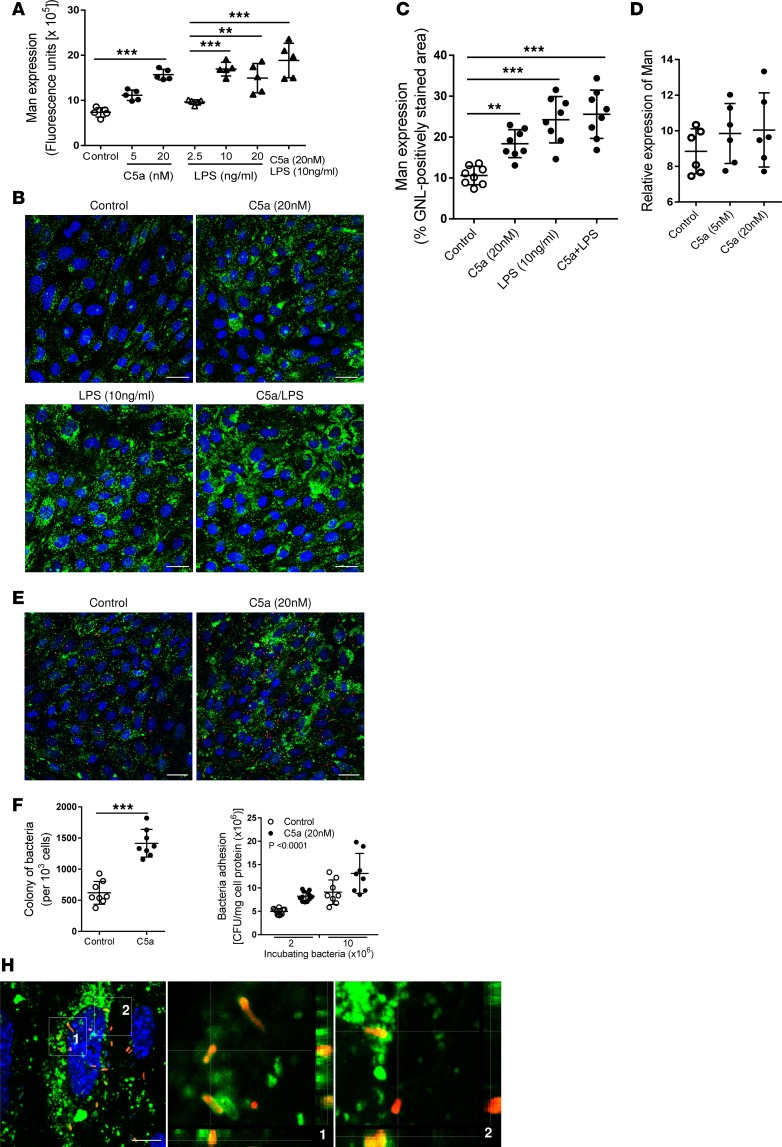
C5a stimulation upregulates mannosyl residue expression and enhances bacterial adhesion in renal tubular epithelial cells (in vitro). (**A**) Effect of C5a and/or LPS stimulation (for 24 hours) on mannosyl residue (Man) expression in primary cultured renal tubular epithelial cells (RTECs) was examined by fluorescence intensity–based microplate assay. Data were analyzed by 1-way ANOVA with Tukey’s multiple comparisons test (*n* = 5 individual wells). **P < 0.005, ***P < 0.001. (**B**) Representative fluorescence images of Man expression in control or C5a and/or LPS pretreated RTECs (nonpermeabilized). Man (green) detected by fluorescein-labeled *Galanthus* *nivalis* lectin (GNL) and DAPI (blue) are shown. Scale bars: 25 μm. (**C**) Quantification of Man expression corresponding to the images shown in **B**. Data were analyzed by 1-way ANOVA with Tukey’s multiple comparisons test (*n* = 8 coverslips per group, under ×630 magnification). *P < 0.05, ***P < 0.001. Data shown are pooled from 4 individual experiments. (**D**) Quantification of Man expression in RTECs prepared from *C5aR1^–/–^* mice assessed by fluorescence microscopic analysis following C5a stimulation (*n* = 6 coverslips per group, under ×630 magnification). Data shown are pooled from 3 individual experiments. (**E**) Representative fluorescence images of bacterial adhesion to RTECs that had been pretreated with or without C5a for 24 hours, then incubated with TRITC-labeled J96 (1 × 10^7^/well) for 1 hour. *E*. *coli* (red), Man (green), and DAPI (blue) are shown. Scale bars: 25 μm. (**F**) Quantification of bacteria corresponding to the images shown in **E**. Data shown are from 8 individual images (under ×630 magnification, from 3 coverslips) per group and representative of 3 independent experiments. ***P < 0.001, analyzed by unpaired 2-tailed Student’s *t* test. (**G**) Bacterial adhesion to RTECs evaluated by CFU assay. Data were analyzed by 2-way ANOVA (*n* = 8 individual wells and representative of 3 independent experiments). ***P < 0.001. (**H**) Confocal microscopic images of bound bacteria in RTECs (nonpermeabilized) that had been incubated with labeled J96 for 1 hour. Bacteria (red), Man (green), and DAPI (blue) are shown. Left image: Compressed image. Scale bars: 10 μm. Middle and right images corresponding to the boxed regions in the left image show the cross-sectional views in *Z*-stack (bottom and side panel) of RTECs, Man, and bacteria, demonstrating association of Man and J96 at the cell surface of RTECs. A representative of 3 experiments is shown.

We considered that C5a/C5aR1 interactions may contribute to the mass of Man on the luminal surface of renal tubules and promote bacterial adhesion/colonization. How C5a/C5aR1 interactions influence both Man expression and bacterial adhesion was examined using primary RTEC cultures. Following C5a and/or LPS stimulation of RTECs, expression of Man was assessed by fluorescence intensity–based microplate and fluorescence microscopy. Results obtained from both assays indicate that C5a or LPS stimulation alone increased surface expression of Man. Combined, C5a/LPS stimulation induced more Man expression than C5a treatment alone (Figure 4, A–C). Notably, the stimulatory effect of C5a on Man expression was absent in RTECs from *C5aR1^–/–^* mice (Figure 4D), supporting that C5a stimulates Man expression through engagement of C5aR1. Confocal microscopy and bacterial plate count assays revealed that pretreatment of RTECs with C5a enhanced bacterial adhesion to RTECs (Figure 4, E–G). The association of bacteria with surface Man on RTECs was shown by confocal *Z*-stack images (Figure 4H). These observations support the concept that C5a/C5aR1 interactions upregulate Man expression in RTECs and facilitate Man-dependent UPEC adhesion.

**Figure 5:**
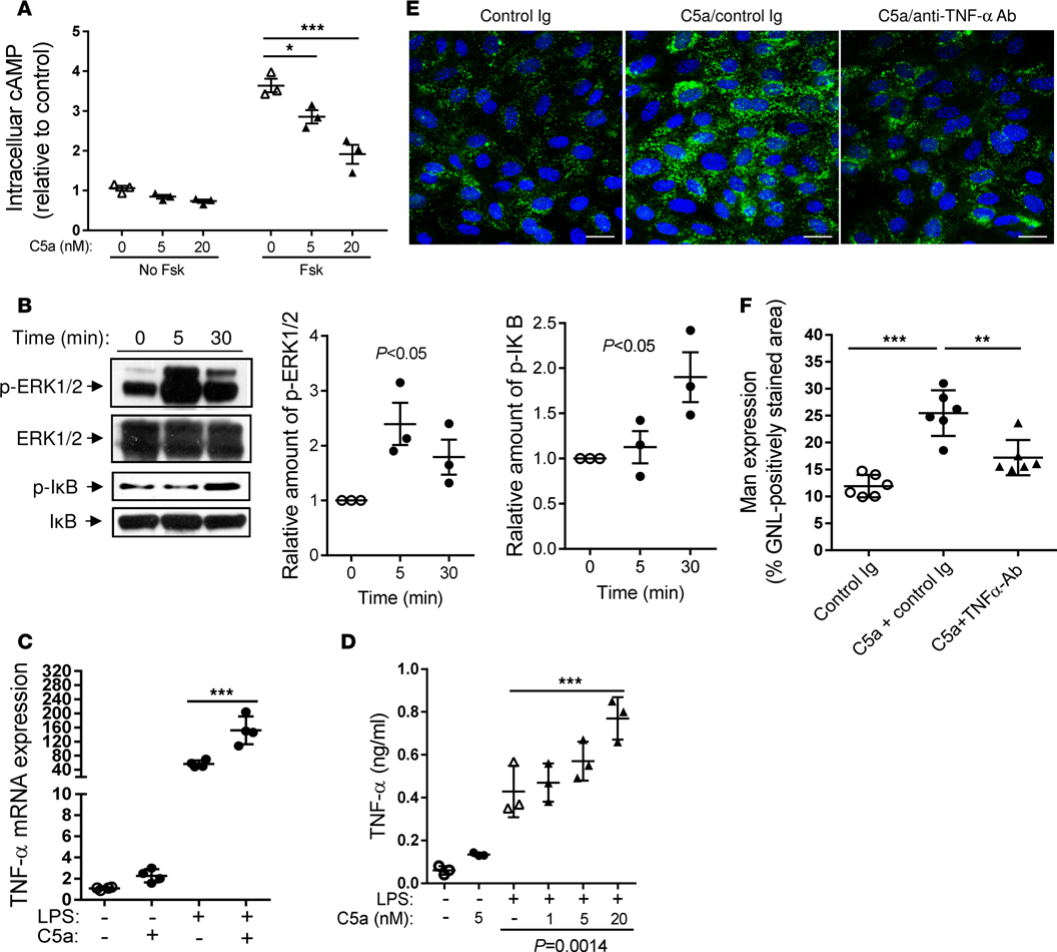
Effects of C5a on intracellular signalling, proinflammatory cytokine production, and mannosyl residue expression in renal tubular epithelial cells (in vitro). (**A**) Renal tubular epithelial cells (RTECs) cultured from WT mice were preincubated with or without forskolin (Fsk, a cAMP elevating agent) (10 μM) for 1 hour and stimulated with C5a (0–20 nM) for 30 minutes. Intracellular cAMP levels were measured in the cell lysates, shown as changes relative to control (untreated cells). *n* = 3/group, resulting from 3 independent experiments. **P < 0.005. (**B**) Western blot analysis for ERK1/2 and IκB phosphorylation in RTECs after C5a (20 nM) stimulation for up to 30 minutes. In each set of blots, the top row of bands corresponds to membrane incubated with appropriate anti-phospho antibody and the bottom row of bands corresponds to membrane incubated with appropriate total antibody. Relative amounts of protein phosphorylation are shown in the right panel of each set of blots. *n* = 3/group, resulting from 3 independent experiments. (**C**) Relative mRNA levels of TNF-α in the RTECs, determined by RT-qPCR. *n* = 4/group, resulting from 4 independent experiments. ***P < 0.001. (**D**) Secretion of TNF-α by the RTECs (supernatants from 24-hour cultures), measured by ELISA. *n* = 3/group, resulting from 3 independent experiments. ***P < 0.001; *P* = 0.0014 (LPS versus LPS plus C5a treatment). (**E**) Representative images of Man (green) in RTECs that had been pretreated with control Ig, C5a/control Ig, or C5a/anti–TNF-α antibody for 24 hours. Scale bars: 25 μm. (**F**) Quantification of Man expression in RTECs in **E**. *n* = 6 coverslips/group, resulting from 3 independent experiments. **P < 0.005, ***P < 0.001. Data were analyzed by 1-way ANOVA with Tukey’s multiple comparisons test (**A**, **C**, **D**, and **F**) or 1-way ANOVA (**B**).

Having demonstrated that C5a has a stimulatory effect on Man expression in RTECs, we sought to identify the signal transduction pathways responsible for the action of C5a. Binding of C5a to C5aR1 triggers a variety of intracellular signaling pathways including cAMP, ERK1/2, and NF-κB, and mediates a number of cellular processes in different types of cells (32–34); however, little is known in RTECs. In addition, activation of inflammatory signaling ERK1/2 and NF-κB has been suggested as an important mechanism involving the regulation of surface N-glycans in endothelial cells (27). To investigate whether the effect of C5a/C5aR1 interactions on Man expression in RTECs is dependent on such a mechanism, we examined the influence of C5a on intracellular signaling within RTECs, specifically cAMP/PKA/CREB, ERK, and IκB (a catalytic subunit of NF-κB essential for NF-κB activation). C5a stimulation significantly reduced intracellular levels of cAMP (a pivotal second messenger with a broad range of antiinflammatory activities) in RTECs, which was more pronounced by pretreatment of RTECs with forskolin (a cAMP elevating agent) (Figure 5A). Accordingly, the phosphorylation of PKA and CREB (the downstream signaling of cAMP) in RTECs was reduced by C5a stimulation (Supplemental Figure 4). By contrast with cAMP/PKA/CREB, the phosphorylation of ERK and IκB (which plays a critical role in mediating proinflammatory responses) was increased by C5a stimulation (Figure 5B). In addition, we examined the effects of C5a on TNF-α production in RTECs. RT-qPCR and ELISA results showed that in the presence of LPS, C5a upregulated both gene expression and the release of TNF-α by RTECs (Figure 5, C and D). We then assessed whether TNF-α is responsible for C5a action on Man upregulation. Addition of anti–TNF-α antibody in RTEC cultures significantly suppressed C5a-mediated upregulation of Man expression (Figure 5, E and F). Taken together, these results led to the suggestion that C5a upregulates Man expression in RTECs, at least in part, through the effects of TNF-α driven by C5aR1-mediated intracellular signaling.

### Relative contributions of C5aR1 on parenchymal and infiltrating cells to the development of APN.

**Figure 6:**
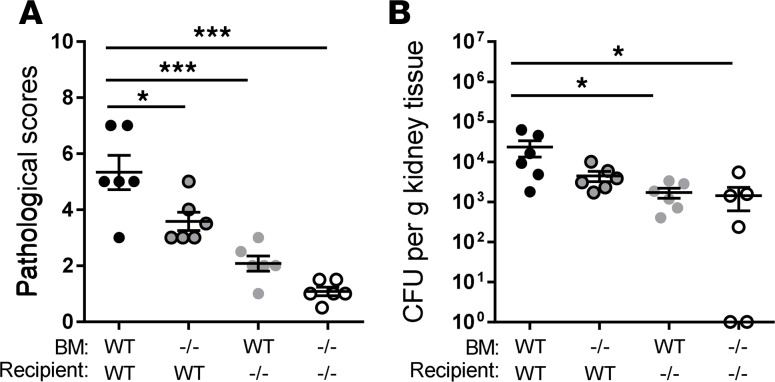
Relative contributions of C5aR1 on parenchymal and infiltrating cells to the development of acute pyelonephritis. (**A**) Histological scores of kidney sections of 4 groups of chimeric mice at 48 hours postinfection (hpi). (**B**) Bacterial loads in the kidney tissues corresponding to the 4 groups of mice in **A**. Each data point represents an individual mouse; small horizontal lines indicate the mean. All data were analyzed by 1-way ANOVA with Tukey’s multiple comparisons test (*n* = 6 mice/group). *P < 0.05, ***P < 0.001. BM, bone marrow; WT, wild-type littermates; –/–, *C5aR1^–/–^* mice.

To evaluate the contribution of C5aR1 on parenchymal and/or infiltrating cells to the development of infection, we induced UTI in 4 groups of chimeric mice generated by bone marrow transplantation of irradiated recipient mice (denoted as *WT*→*WT*, *C5aR1^–/–^*→*WT*, *WT*→*C5aR1^–/–^*, *C5aR1^–/–^*→*C5aR1^–/–^*). Attenuated renal bacterial load and tissue damage were observed in mice lacking C5aR1 on recipient renal cells (*WT*→*C5aR1^–/–^*) or donor myeloid cells (*C5aR1^–/–^*→*WT*), compared with mice that express C5aR1 on both compartments (*WT*→*WT*), at 48 hpi. Mice lacking C5aR1 on renal parenchymal cells were afforded better protection, as the reduction of tissue damage and bacterial load was more pronounced in this group of mice (Figure 6).

### Blocking C5aR1 reduces Man expression and bacterial colonization of renal tubular epithelium, and protects mice from APN.

**Figure 7:**
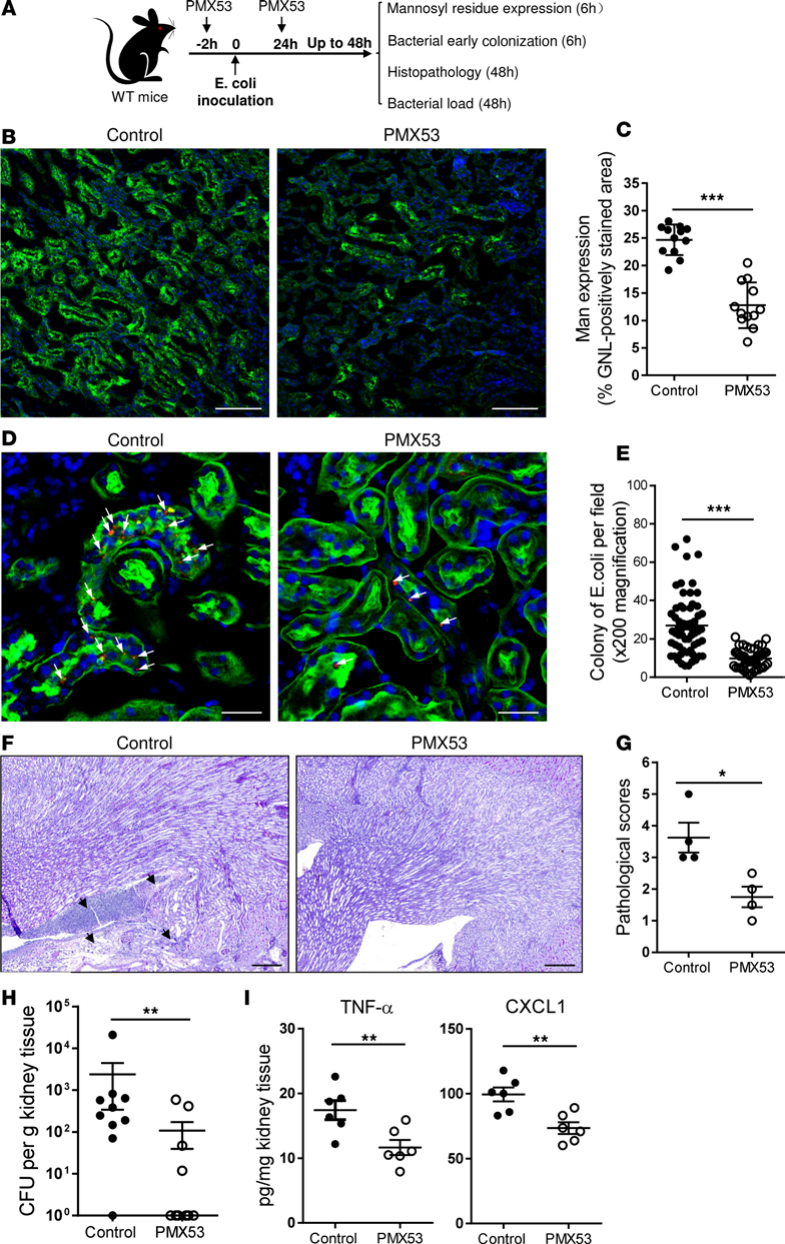
Blocking C5aR1 reduces mannosyl residue expression and bacterial colonization of renal tubular epithelium, and protects mice from acute pyelonephritis. (**A**) A schematic diagram of the experimental design. WT mice received control or PMX53 (1 mg/kg) treatment 2 hours before bacterial inoculation and 24 hours after inoculation. (**B**) Representative images of mannosyl residues (Man) (green) in kidney sections of infected WT mice that received control or PMX53 (1 mg/kg) treatment at 6 hours postinoculation. Scale bars: 100 μm. (**C**) Quantification of Man expression in renal tubules corresponding to the mice in **B**. Data were analyzed by unpaired 2-tailed Student’s *t* test (*n* = 12 viewing fields from 4 mice/group). (**D**) Representative images showing early bacterial colonization of renal tubular epithelium in control or PMX53-treated mice. TRITC-labeled J96 (red), proximal tubular marker (L-fucose) detected by *Lotus* *tetragonolobus* lectin (LTL) (green), and nuclear marker DAPI (blue) are shown. Arrows indicate bacterial colonies. Scale bars: 30 μm. (**E**) Quantification of bacterial colonies in renal epithelium corresponding to control or PMX53-treated mice in **D**. Data were analyzed by unpaired 2-tailed Student’s *t* test (*n* = 60 viewing fields from 6 mice/group). (**F**) Representative images of PAS staining of kidney sections of control or PMX53-treated mice at 48 hours postinoculation. Arrows indicate the lesions. Scale bars: 250 μm. (**G**) Histological scores of kidney sections of control or PMX53-treated mice in **F** (*n* = 4 mice/group). In **F** and **G**, a representative of 2 experiments is shown. (**H**) Bacterial loads in kidney tissues of control or PMX53-treated mice determined by CFU assay (*n* = 10 mice/group). Data shown are pooled from 2 individual experiments. (**I**) Intrarenal TNF-α and CXCL1 levels in control or PMX53-treated mice evaluated by ELISA (*n* = 6/group). (**G**–**I**) Each data point represents an individual mouse. Data were analyzed by Mann-Whitney test (**H**) or unpaired 2-tailed Student’s *t* test (**G** and **I**). *P < 0.05, **P < 0.005, ***P < 0.001.

To explore the therapeutic potential of targeting C5aR1 in UTI, we employed PMX53 (a recognized C5aR1 antagonist) (35) in our APN model. PMX53 treatment significantly reduced both Man expression and bacterial colonization in renal tubular epithelium (Figure 7, A–E). A concomitant reduction in pathological damage, bacterial load, and intrarenal production of proinflammatory mediators was observed in the PMX53-treated mice compared with the control group (Figure 7, F–I). The results were consistent with the knockout mouse studies, confirming a role of C5aR1 in the development of APN.

## Discussion

Our recent work in a murine model of CPN has demonstrated a pathogenic role for C5aR1 in chronic renal inflammation and tubulointerstitial fibrosis and suggested the underlying mechanisms. These mechanisms are involved in C5a/C5R1 signaling–mediated upregulation of intrarenal production of proinflammatory and profibrogenic mediators and impairment of MΦ killing function, which lead to persistent infection and renal scaring (20). The present study focuses on a murine model of APN. The findings not only demonstrate a previously unknown role for C5aR1 in the pathogenesis of APN, but also describe a potentially novel mechanism by which C5aR1 contributes to ascending UTI. This involves both upregulation of Man expression and promotion of early bacterial colonization of renal tubular epithelium, thereby increasing susceptibility to ascending UTI.

Man are the well-characterized carbohydrate ligand for FimH of type 1 fimbriae on *E*. *coli*. It has been suggested that the interactions of type 1 fimbriae and mannose-containing host receptors in uroepithelium is an important pathogenic mechanism for bladder infection (12, 24, 26). However, the presence and distribution of such ligands in renal tubular epithelium, binding of UPECs to ligands within the kidney, and the contribution of the ligands to renal infection are largely unknown. As virtually all clinical UPEC isolates express type 1 fimbriae (36), addressing these issues is important for improving our understanding of the pathogenesis of ascending UTI. In the present study, we clearly show that Man (a known ligand for type 1 fimbriated UPECs) are expressed within renal tract epithelium and on cultured RTECs. In addition, we found that C5aR1 deficiency is associated with reduced levels of Man expression on the luminal surface of renal tract epithelium. Receptor deficiency curtails early bacterial colonization, providing protection from ascending UTI. Thus, our data support the argument that C5a/C5aR1–associated upregulation of Man on renal tubules contributes to renal infection through interactions with type 1 fimbriae. In agreement with the role of type 1 fimbriae in renal infection, a previous study has shown that type 1 fimbriae are required for UPEC adhesion and colonization in the kidney in vivo (37). In addition to the role of C5aR1 in kidney infection, in separate studies, we have found that *C5aR1^–/–^* mice had also reduced bladder infection, in both chronic and acute models of UTI (20) (Wu et al., unpublished observations), suggesting a pathogenic role for C5aR1 in bladder infection. Given the critical role of the bladder infection in ascending UTI, C5aR1-associated bladder infection could potentially contribute to renal infection.

To address the mechanisms that C5aR1 employs to influence Man expression in the renal tract epithelium, we assessed the possibility that C5a/C5aR1 interactions mediate upregulation of Man expression in renal tract epithelium. We detected the expression of C5aR1 in renal tract epithelium and the presence of C5a in both kidney tissue and urine, and C5aR1 and C5a upregulation following infection. Therefore, arguably, it is possible that C5a/C5aR1 interactions occur in renal tract epithelium under normal conditions, thereby mediating the tubule cell activation. This in turn leads to an upregulation of Man expression. Lectin staining of renal tissue demonstrated that absence of C5aR1 specifically reduced Man expression in both noninfected and infected mice, reflecting a requirement for C5a/C5aR1 interactions for Man expression in both normal and pathogenic conditions. The notion that C5a/C5aR1 interactions mediate upregulation of Man in renal tubular epithelium was supported by our in vitro findings where C5a stimulation, particularly in the presence of LPS, upregulated cell surface Man expression and enhanced UPEC adhesion to RTECs. Further studies revealed that C5a upregulates Man expression on RTECs, at least in part, through an effect of TNF-α driven by C5aR1-mediated intracellular signaling (downregulation of cAMP/PKA/CREB, upregulation of ERK and NF-κB). In addition to the effects of C5a/C5aR1 on downstream signaling, we also considered the direct impact of C5aR1 on bacterial adhesion. Our confocal microscopy revealed a large-scale association between C5aR1 and Man on the luminal surface of the renal tract epithelium. This, together with the fact that C5aR1 is an N-linked glycosylated protein, raises the possibility that C5aR1 as a Man-containing glycoprotein may function as a carbohydrate ligand for UPECs and mediate type 1 fimbriae–dependent bacterial adhesion/colonization.

Chimera studies suggested that C5aR1 on renal cells plays a more prominent role in this model because an absence of C5aR1 on renal cells conferred better protection from infection when compared with an absence of C5aR1 on myeloid cells. The contribution of renal cell C5aR1 in this model may result from epithelial C5aR1–dependent UPEC colonization of renal tract, as discussed earlier. On the other hand, the contribution of inflammatory cell C5aR1 may reflect inflammatory responses driven by C5aR1 signaling, which lead to tissue destruction and upregulation of Man expression in renal tubular epithelium.

Chemical blockade of C5aR1 with the antagonist PMX53 provided protection from APN in this model. One explanation for this effect could be that targeting C5aR1 on epithelial cells inhibits C5a-mediated upregulation of Man expression and subsequent bacterial adhesion/colonization. Additionally, targeting C5aR1 on inflammatory cells would attenuate C5a-mediated proinflammatory responses, consequently reducing tissue damage and curtailing upregulation of Man via proinflammatory mediators in renal epithelium. In agreement with this, the anti-C5 antibody (BB5.1), which prevents the generation of C5a and C5b-9, was found to be effective in conferring protection from APN in this model (data not shown). Therefore, the effects of C5aR1 antagonism in this model could be attributed to the blockade of C5a/C5aR1 interactions on both renal epithelial cells and inflammatory cells.

**Figure 8:**
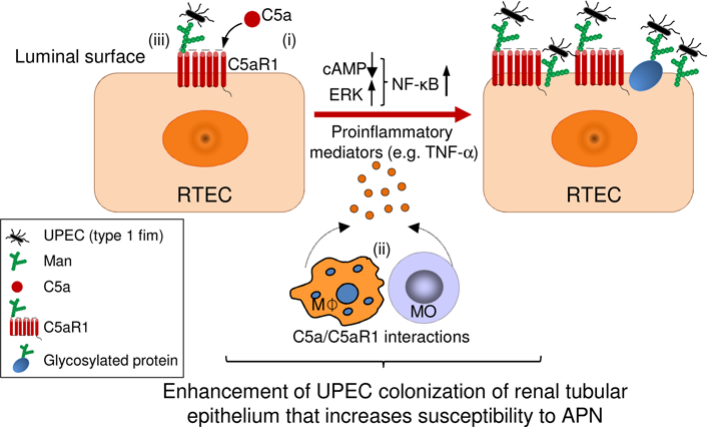
Proposed mechanisms by which C5aR1 enhances UPEC colonization of renal tubular epithelium that increases susceptibility to acute pyelonephritis. Based on our findings in this study, we propose that (i) engagement of C5aR1 with C5a in renal tubular epithelial cells (RTECs) mediates the production of proinflammatory mediators, which results in enhancement of mannosyl residue (Man) expression at the luminal surface of renal tubular epithelium (possibly resulting from Man on the cell membrane or specific glycosylated proteins, including C5aR1), subsequently facilitating type 1 fimbriae–mediated uropathogenic *E*. *coli* (UPEC) adhesion. (ii) This process could be reinforced by proinflammatory mediators produced by inflammatory cells driven by C5a/C5aR1 signaling (downregulation of cAMP, upregulation of ERK and NF-κB). (iii) In addition, C5aR1 on renal tubular epithelium may function as carbohydrate ligands for UPEC binding. All these processes contribute to enhancement of UPEC colonization of renal tubular epithelium that increases susceptibility to acute pyelonephritis (APN).

Based on the findings presented in this study, we propose a potentially novel mechanism by which C5aR1 promotes the development of APN, namely, that C5aR1 mediates enhancement of bacterial adhesion/colonization of renal tubular epithelium. This is achieved through the following processes: (a) engagement of C5aR1 with C5a on RTECs mediates the production of proinflammatory mediators, which results in enhancement of Man expression on the luminal surface of the renal tubular epithelium, subsequently facilitating type 1 fimbriae–mediated UPEC adhesion; (b) C5a/C5aR1 signaling promotes the secretion of proinflammatory mediators by inflammatory cells; and (c) C5aR1 expressed on the renal tubular epithelium functions as a carbohydrate ligand for UPEC binding (Figure 8). Therefore, C5a/C5aR1–mediated early inflammatory responses (in response to initial infection) in the kidney facilitate bacterial adhesion/colonization of renal tubular epithelium. This results in further amplification of inflammatory responses, consequently causing more tissue damage and UPEC colonization.

In conclusion, the present study is the first to our knowledge to demonstrate a pathogenic role for C5aR1 in APN and address the underlying mechanisms of C5aR1 promoting the development of APN. Our study provides insight into the mechanisms of bacterial–host cell carbohydrate interactions facilitating tissue invasion. Furthermore, the findings presented in this study, coupled with the findings of our previous study in CPN, highlight pathogenic roles for C5aR1 in ascending UTI and opens up a new avenue for therapeutic targeting in UTI.

## Methods

### Materials.

We used the following reagents and materials: monoclonal anti–mouse CD45 (30-F11, APC), Ly6G (1A8, PE), Ly6C (HK1.4, PE/Cy7), CD11b (M1/70, FITC) (all BioLegend); polyclonal goat anti–mouse C5aR1 (M-19) (Santa Cruz Biotechnology); fluorescein-labeled *Lotus* *tetragonolobus* lectin (LTL), -GNL, -LCA, and -SNA (all Vector Laboratories); polyclonal rabbit anti–mouse cytokeratin (wide spectrum) (Abcam); cell culture medium, fetal calf serum, insulin-transferrin-selenium solution, gentamicin, and CountBright absolute counting beads (Life Technologies Ltd); 4′,6-diamidino-2-phenylindole (DAPI) (Life Sciences); Alexa Fluor 488 donkey anti–goat IgG (Jackson ImmunoResearch Lab Inc.); FcR-blocking antibody (CD16/32, 2.4G2) and mouse ELISA kits for TNF-α and paired ELISA antibodies for C5a (BD Biosciences); MPO assay kit and mouse ELISA kit for CXCL1, recombinant mouse TNF-α, monoclonal anti–mouse TNF-α (MP6-XT22), and isotype control (43414) (R&D Systems); hydrocortisone, triiodothyronine, thioglycollate, tetramethylrhodamine (TRITC), protease inhibitor cocktail, LPS (from *E*. *coli* with serotype O55:B5, contains *O* antigen) (Sigma-Aldrich); collagenase D (Roche); collagenase type II (Worthington Biochemical Corp.); C5aR1 peptide antagonist (PMX53, Ac-Phe-cyclo [Orn-Pro-dCha-Trp-Arg]) and control peptide (random sequence) (synthesized by GenScript).

### Mice.

Homozygous *C5aR1^–/–^* mice were generated by homologous recombination in embryonic stem cells (38) (provided by Bao Lu and Craig Gerard, Harvard Medical School, Boston, Massachusetts, USA) and backcrossed onto the C57BL/6 (H-2b) parental strain for at least 12 generations. WT littermate mice were used as controls. Female mice (8–10 weeks old) were used in all experiments. All mice were maintained in specific pathogen–free conditions.

### Bacterial strains.

*E*. *coli* J96 (serotype O4; K6), a human pyelonephritis isolate, was provided by Rodney Welch (University of Wisconsin, Madison, Wisconsin, USA). J96 is a serum-resistant, hemolysin-secreting *E*. *coli* strain that expresses both type 1 and P fimbriae. Cystitis isolate NU14 and the isogenic *fimH-*mutant NU14-1 were provided by Scott Hultgren (Washington University School of Medicine, St. Louis, Missouri, USA). IH11128 (075:K5:H–) is a mannose-resistant strain expressing both Dr and type 1 fimbriae but lacking P fimbriae and hemolytic activity (provided by Bogdan Nowicki, University of Texas, Galveston, Texas, USA). CFT073 (O6:H1:K2) expresses both type 1 and P fimbriae (provided by Sivaramesh Wigneshweraraj, Imperial College, London, UK).

### Induction of pyelonephritis.

Murine UTI, a model of ascending UTI leading to pyelonephritis, was induced in female mice by bladder inoculation with UPEC (2 × 10^8^ CFU in 50 μl PBS) via urethra as previously described (28, 29). Mice were killed at different time points (up to 72 hpi) for evaluation of renal histopathology and bacterial load. In some experiments, mice were treated with C5aR1 peptide antagonist (PMX53) (35) or control peptide (1 mg/kg), at 2 hours before and 24 hours after inoculation by intraperitoneal (i.p.) injection.

### Measurement of bacterial load in the kidney.

Total bacterial load in kidney tissue was measured by bacterial plate count assay as previously described, with modifications (19, 39). In brief, the tissue was homogenized in 2 ml (for the kidney) of sterile PBS. Fifty microliters of a serial dilution of homogenates was plated on duplicate CLED plates. After incubation of plates for 24 hours at 37°C, bacterial CFU on the plates were manually counted and expressed as an average CFU per gram of kidney tissue.

### Assessment of kidney histopathology.

Kidneys were fixed in a solution of 4% formalin in PBS for 24 hours and embedded in paraffin. Paraffin sections (3 μm) were stained with periodic acid–Schiff (PAS) or hematoxylin and eosin (H&E). The severity of renal histopathology (i.e., tissue destruction, cellular infiltration, bacterial patchiness, and presence of abscesses), was graded using a 7-point scale as described previously with modifications (27, 40), in which 0, 1, 2, and 3 indicated normal, mild, moderate, and severe pyelonephritis, respectively (pathological changes were mainly located within the cortical-medullar junction and the medulla); while 4, 5, and 6 indicated mild, moderate, and severe pyelonephritis, respectively (pathological changes spread to more parts of the kidney). The assessment was performed in a blinded fashion by 2 persons. Two to 3 kidney sections of each mouse were viewed and are presented as an average score.

### Lectin staining in kidney tissue.

Frozen sections of kidney were stained with fluorescein-labeled lectin (GNL or LCA and SNA) (20 μg/ml in PBS) and DAPI, and then viewed and imaged with a confocal laser scanning microscope system (Leica TCS SP8). The percentage of positive staining area in each image under ×200 magnification was calculated by using ImageJ software v1.41 (NIH). Three to 4 viewing fields at the cortical-medullar junction for each kidney were examined.

### Assessment of bacterial colonization in kidney tissue.

Overnight cultures of UPEC strains were washed and then suspended in PBS (10^9^ CFU/ml). TRITC was added to a final concentration of 1 mg/ml and incubated for 3 hours with gentle shaking in the dark. Bacteria were vigorously washed to remove unbound TRITC. For detection of bacterial colonization in kidney tissues, TRITC-labeled *E*. *coli* J96 (2 × 10^8^ CFU, in 50 μl PBS) were injected into the bladder via urethra. Mice were killed at 6 hpi, frozen sections of infected kidney were stained with DAPI (for nuclei) and LTL (for proximal tubular cells), and then viewed and imaged with the Leica SP8 system. Bacterial colonies were manually counted at ×200 magnification and results are expressed as number of colonies per field. Six to 10 viewing fields, randomly selected from inner medullar, cortical-medullar junction, and outer cortex (2–4 fields for each area) for each kidney were examined.

### UPEC in situ binding to kidney tissue.

Frozen sections (4 μm) of OCT-embedded kidneys were immobilized on Superfrost Plus microscope slides (Thermo Fisher Scientific) and were rehydrated in PBS for 5 minutes and incubated with medium containing 0.1% BSA for 1 hour, and then with TRITC-labeled UPECs (2 × 10^8^ CFU, in 200 μl of PBS containing 0.1% BSA) for 1 hour at 37°C. Sections were then gently washed 5 times to remove unattached bacteria and stained with DAPI and fluorescein-labeled GNL, and then viewed and imaged with the Leica SP8 system. Bacterial colonies were manually counted at ×200 magnification and results were expressed as number of colonies per field. Five viewing fields at the cortical-medullar junction for each kidney section were examined.

### Assessment of inflammatory cell infiltration in the kidney.

Single renal cell suspensions were prepared using a method described previously, with modifications (19, 41). Kidneys were weighed, minced, and incubated with collagenase D (0.75 mg/ml) for 10 minutes at 37°C with gentle agitation. The collagenase was inactivated with an equal volume of DMEM-F12 containing 10% FCS. The digested tissue mixture was then passed through a 40-μm nylon sieve to remove tissue debris. The cell segments were collected and treated with RBC lysis buffer to remove remaining RBC. The cell pellet was washed and resuspended in PBS containing 1% BSA and followed by flow cytometric analysis. The cells were preincubated with FcR-blocking antibody (CD16/32), then stained with rat anti–mouse APC-conjugated CD45, PE-conjugated Ly6G, PEcy7-conjugated Ly6C, and FITC-conjugated CD11b antibodies, or the appropriate isotype control antibodies at 4°C for 20 minutes. In order to quantify absolute cell counts in kidney tissue, we used CountBright absolute counting beads in our flow cytometry assays, according to the manufacturer’s instructions. All flow cytometric analysis was performed using a FACSCalibur flow cytometer (BD Biosciences) and FlowJo software (Tree Star).

### Generation of chimeric mice.

Chimeric mice were generated using a method established in our laboratory (42). Mice were first irradiated with a dosage of 9 Gy and reconstituted with an intravenous administration of 7.5 × 10^6^ donor bone marrow cells within 3 hours after irradiation. At 4 weeks after bone marrow transplantation, chimeric mice were used for induction of pyelonephritis.

### Cell cultures.

Primary RTEC cultures were prepared from kidneys of naive male WT or *C5aR^–/–^* mice as described previously (43). In brief, minced cortex and outer medulla was digested with 0.1% collagenase II and passed through a 40-μm nylon sieve. The cells and tubules were collected and cultured in DMEM-12 medium that contained 2% FCS, insulin (5 μg/ml), transferrin (5 μg/ml), selenium (5 ng/ml), hydrocortisone (40 ng/ml), and triiodothyronine (10^–12^ M). Non-passaged, 7- to 10-day-cultured RTECs were used for further experiments.

### Assessment of Man expression in cultured RTECs.

Mannose residue expression in cultured RTECs was assessed by a fluorescence intensity–based microplate assay or fluorescence microscopic analysis. For microplate assay, confluent RTECs grown in 24-well plates were incubated with or without C5a and/or LPS for 24 hours at 37°C, and then with fluorescein-labeled GNL (20 μg/ml in PBS) for 1 hour at 37°C. Cells were then vigorously washed and lysed with sterile H_2_O. The fluorescence in the lysate was measured using a fluorescence plate reader (SpectraMax i3, Molecular Devices) with an excitation wavelength of 490 nm and results expressed as relative fluorescence units. Fluorescence microscopic analysis was used to detect surface Man on RTECs. RTECs grown on the coverslips were pretreated with C5a and/or LPS and stained with fluorescein-labeled GNL and DAPI. Images were taken with the Leica SP8 system under ×630 magnification. The percentage of positive staining area in each image was calculated by using ImageJ software v1.41. Three to 4 viewing fields randomly selected from each coverslip were examined.

### Assessment of bacterial adhesion to cultured RTECs by fluorescence microscopy.

RTEC monolayers grown on coverslips in 24-well plates were incubated with TRITC-conjugated J96 (1 × 10^7^ CFU/well) for 1 hour at 37°C. Cells were vigorously washed to remove unattached bacteria and stained with DAPI and fluorescein-labeled GNL, and then viewed and imaged with the Leica SP8 system. Bound bacteria and RTEC numbers were counted at ×630 magnification, and results are expressed as number of bacteria per 10^3^ tubule cells. Two to 3 viewing fields randomly selected from each coverslip were examined and the average number is presented.

### Assessment of bacterial binding in RTECs by bacterial plate count assay.

The assays were performed as described previously (44). Briefly, confluent RTEC monolayers were incubated with J96 (2 × 10^6^ or 1 × 10^7^ CFU/well) for 1 hour at 37°C. Cells were then vigorously washed to remove unattached bacteria and lysed with sterile H_2_O. The lysate was then plated out on cysteine-, lactose-, and electrolyte-deficient (CLED) agar plates and incubated at 37°C for 24 hours. In some experiments, RTEC monolayers were preincubated with or without C5a for 24 hours and subjected to binding assay. Colonies were counted and results are expressed as CFU/gram of cell protein. RTEC protein concentrations were measured using the Coomassie (Bradford) protein assay kit according to the manufacturer’s instructions.

### RT-qPCR.

Total RNA extraction from kidney tissue and cells and reverse transcription reactions were performed as previously described (45). RT-qPCR was performed using the DyNAmo HS SYBR Green qPCR kit from Thermo Fisher Scientific and an MJ Research PTC-200 Peltier Thermal Cycler from Bio-Rad. Amplification was performed according to the manufacturer’s cycling protocol and tested in duplicate. The relative gene expression was analyzed using the 2^–ΔΔ*C*T^ method (46) and expressed as 2^–ΔΔ^ ^(Ct)^, where Ct is cycle threshold, ΔΔ (Ct) = test samples Δ (Ct) – control sample Δ (Ct); Δ (Ct) = test gene (Ct) – 18S (Ct). The control samples were either normal kidney tissues or untreated cells. The test samples were infected kidney tissues or treated cells. The information for primer sequences is given in Supplemental Table 1.

### ELISA.

Renal cytokine levels were measured in the supernatant of the kidney homogenates by ELISA, using ELISA kits for CXCL1 and TNF-α, according to the manufacturer’s instructions. For preparation of kidney homogenates, individual kidneys were weighed and homogenized in PBS containing 1% Triton X-100, 1 mM EDTA, and 1% protease inhibitor cocktail and then spun clear at 10,000 *g* for 10 minutes as previously described (47). The resulting supernatants were analyzed by ELISA. Mouse C5a levels were measured in urine and kidney homogenates using paired ELISA antibodies for C5a. All test samples were measured in triplicate.

### Statistics.

Data are shown as the mean ± SEM or the readout of individual mice. Mann-Whitney test (for CFU) or unpaired Student’s *t* test was used to compare the means of 2 groups. One-way or 2-way ANOVA was used to compare the means of more than 2 independent groups. All the analyses were performed using GraphPad Prism 7 software. *P* less than 0.05 was considered to be significant.

### Study approval.

At King’s College London, animal experiments were performed in accordance with the Animals (Scientific Procedures) Act of 1986, UK, under a project licence (PPL707854) approved by Home Office, UK. At Xi’an Jiaotong University, animal experiments were performed according to protocols approved by the school Ethics Review Committee for Animal Experimentation.

## Author contributions

WZ and KL conceived and designed the study, supervised the project, and wrote the manuscript. KL, KYW, WW, NW, TZ, NC, YS, CAF, LM, and LLW performed experiments. KL, WZ, WW, KYW, ZYD, and XD analyzed data. EQL, ZFL, and SHS contributed to interpretation of results and helped with experimental design.

## Supplementary Material

Supplemental data

## Version 1 12/21/2017

Electronic publication
