# Supplementary material for: C5aR1 promotes acute pyelonephritis induced by uropathogenic E. coli
Source: JCI Insight. 2017 Dec 21;2(24):e97626. doi: 10.1172/jci.insight.97626 (PMC5752266; doi:10.1172/jci.insight.97626)
Supplement: Supplemental data [file jciinsight-2-97626-s001.pdf]

sFigure 1. Stepwise gating strategy used in flow cytometric analysis of cellular infiltration in kidney tissues.

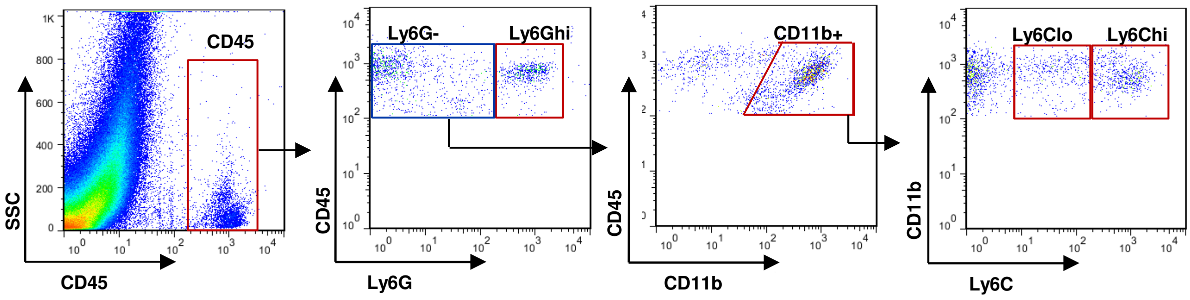

Renal inflammatory cell infiltration was analysed in infected WT and *C5aR1-/- mice* at 24 hpi by flow cytometry. Stepwise gating strategy was used in the flow cytometric analysis of leukocytes (CD45+), neutrophils (CD45+Ly6G+), MO/MΦ (Ly6G-CD11b+), and Ly6Chi MO/MΦ in kidney tissues.

sFigure 2. Expression and distribution of mannosyl residues in normal kidney

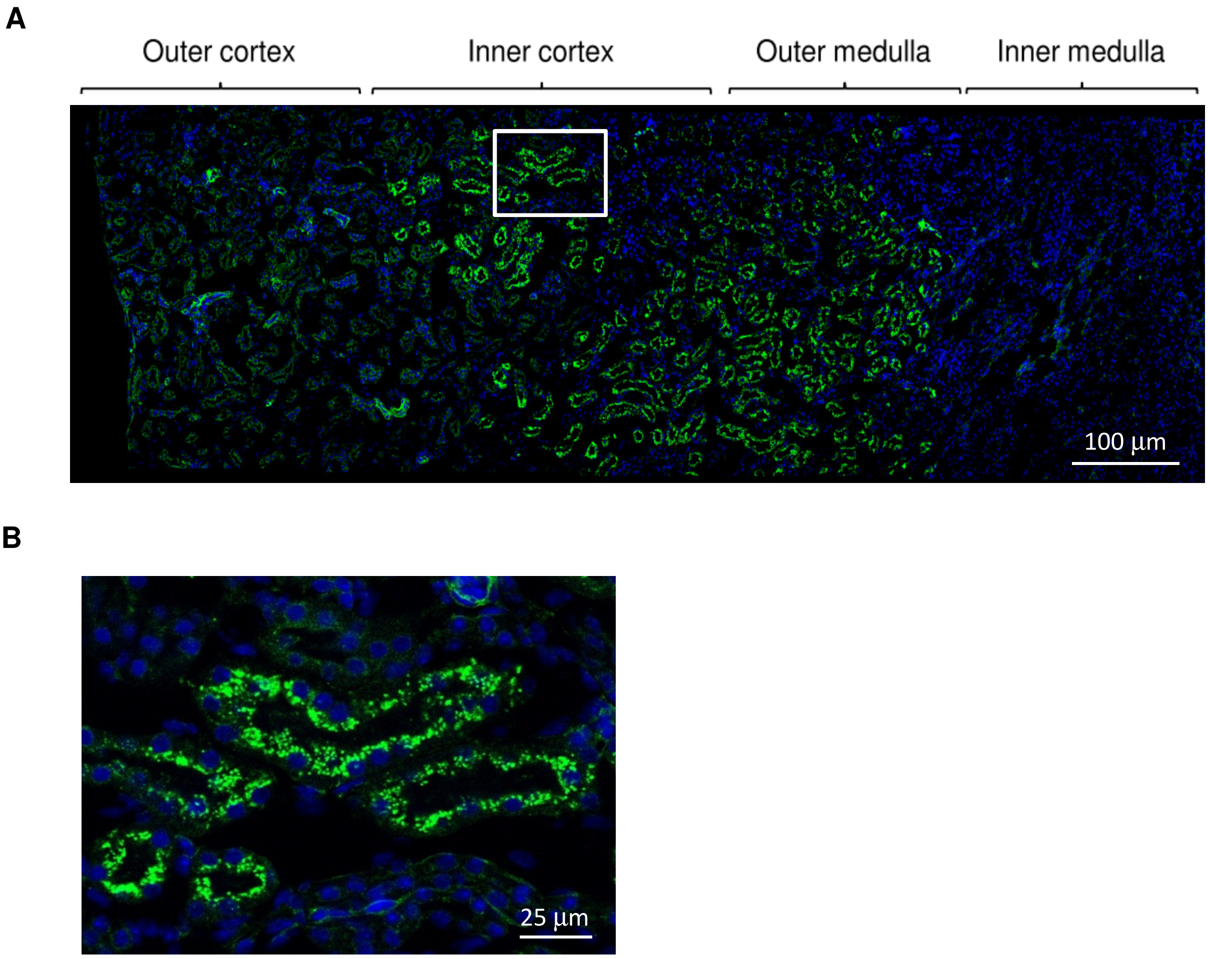

Fluorescence microscopy of normal wild type mouse kidney showing specific detection of mannosyl residues (green) by fluorescein-labelled Galanthus nivalis lectin and counterstaining of nuclei with DAPI (blue). Mannosyl residues were mainly detected in the cortical and medullar tubules **(A)** and predominantly localised at the luminal surface of tubular epithelial cells **(B)**.

sFigure 3. Other glycosylated molecule expression in the kidney of WT and C5aR1-/- mice

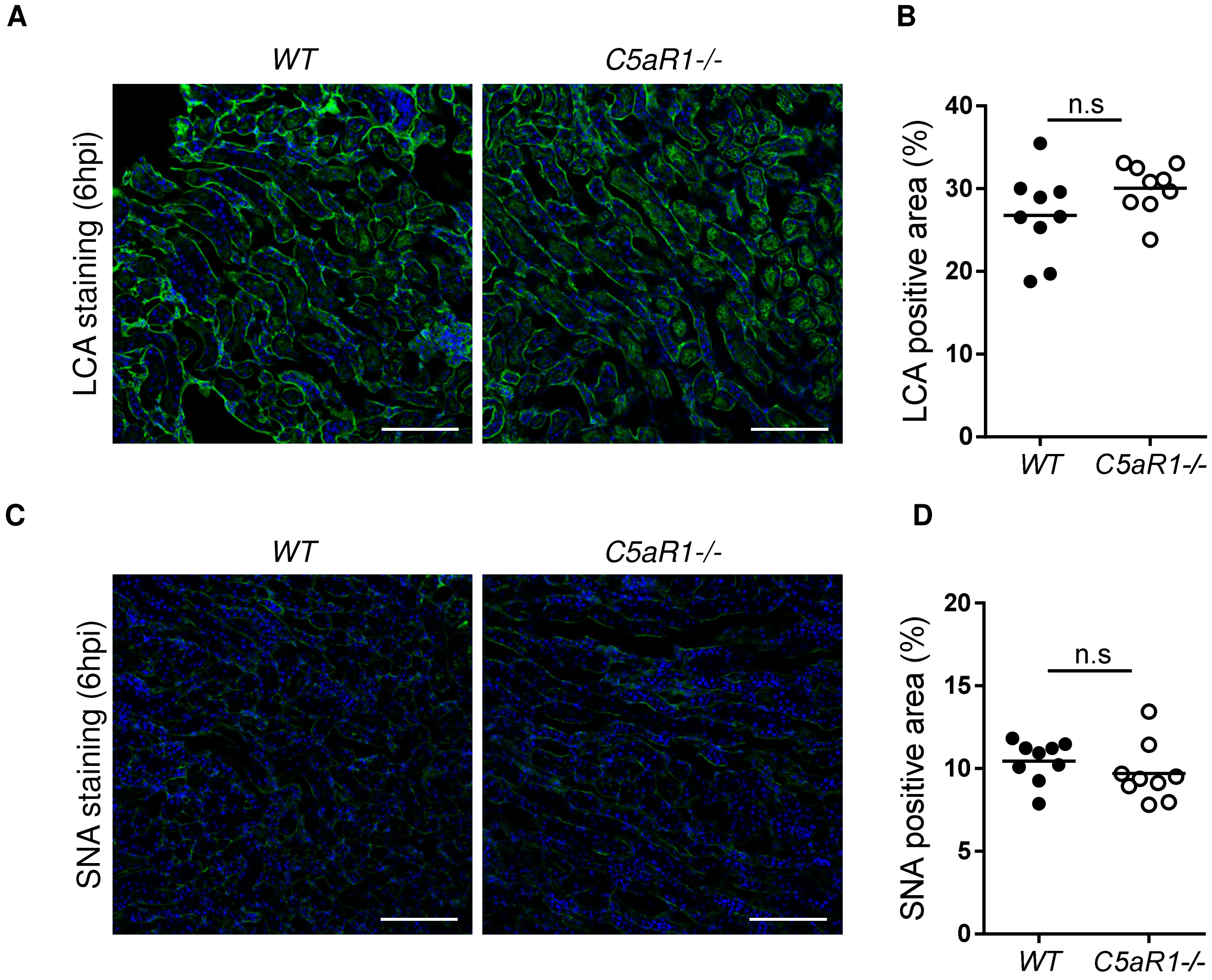

(**A, C**) Representative images of kidney sections of WT and *C5aR1*$^{-/-}$ mice (i.e. infected with J96 for 6 h) stained with Lens culinaris agglutinin (LCA) and Sambucus nigra bark lectin (SNA) for detection of branched fucose/mannose residues and sialic acid attached to terminal galactose, respectively. Scale bars, 100 µm. (**B, D**) Quantification of positively stained area corresponding to the WT and *C5aR1*$^{-/-}$ mice in **A** and **C**. Data were analysed by Unpaired Student's *t* test (9 viewing fields from 3 mice/group). A representative of two experiments is shown.

sFigure 4. C5a stimulation reduces the downstream signalling of cAMP in RTEC

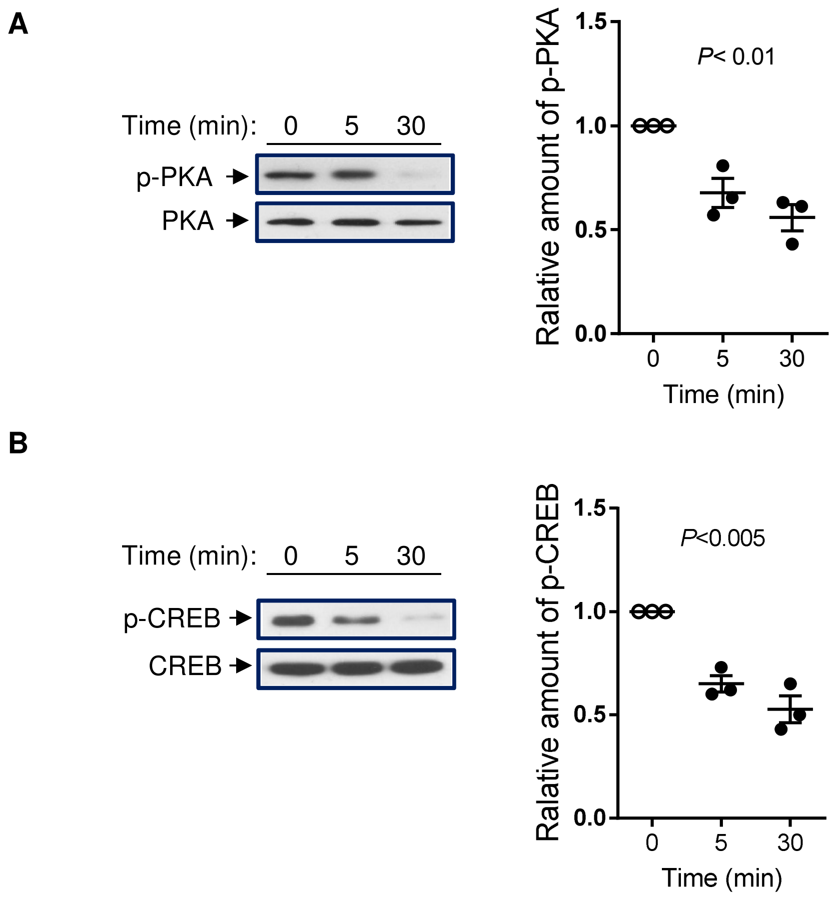

RTEC cultured from WT mice were stimulated with C5a (20nM) up to 30 minutes. Western blot analysis for the downstream signalling of cAMP: (**A**) protein kinase A (PKA) and (**B**) cAMP response element-binding protein (CREB) phosphorylation in RTEC. In each set of blots, the top row of the bands corresponds to incubating membrane with appropriate anti-phospho-antibody and the bottom row of the bands corresponds to incubating membrane with appropriate total antibody. Relative amounts of protein phosphorylation are shown in the right panel of each set of blots. Data were analysed by One-way ANOVA (n=3 resulting from three independent experiments).

Supplementary Table 1

PCR primer sequences and product sizes

Primer*	**Oligonucleotide Sequence**	**Product Size (bp)**	**Gene bank code**
18S-1	5’-ATCCCTGAGAAGTTCCAGCA-3’	153	NM_011296.1
18S-2	5’-CCTCTTGGTGAGGTCGATGT-3’		
TNF-α-1	5’-TGAGCACAGAAAGCATGATCC-3’	200	NM_013693.3
TNF-α-2	5’-GCCATTTGGGAACTTCTCATC-3’		
CXCL1-1	5’-TGAAGCTCCCTTGGTTCAGA-3’	361	NM_008176.3
CXCL1-2	5’-TGCACTTCTTTTCGCACAAC-3’		
CCL2-1	5’-GGCTCAGCCAGATGCAGTTA-3’	219	NM_011333.3
CCL2-2	5’-ATTTGGTTCCGATCCAGGTT-3’		

* Primer-1 is identical to the coding strand; primer-2 is complementary to the coding strand.
